# A wearable electronic based on flexible pressure sensor for running motion monitoring

**DOI:** 10.1186/s11671-023-03788-7

**Published:** 2023-03-01

**Authors:** Xiaoming Chang

**Affiliations:** Physical Education College, Harbin Normal University, Harbin, 150001 Heilongjiang Province China

**Keywords:** Flexible pressure sensor, High linear sensitivity, Wide detection range, Running motion

## Abstract

The flexible pressure sensor is expected to be applied in the new generation of sports wearable electronic devices. Developing flexible pressure sensors with a wide linear range and great sensitivity, however, remains a significant barrier. In this work, we propose a hybrid conductive elastomeric film oxide-based material with a concave-shape micro-patterned array (P-HCF) on the surface that sustainably shows the necessary sensing qualities. To enhance sensing range and sensitivity, one-dimensional carbon fibers and two-dimensional MXene are incorporated into the polydimethylsiloxane matrix to form a three-dimensional conductive network. Micro-patterns with a curved shape in P-HCFs can be able to linear sensitivity across the sensing range by controlling the pressure distribution inside the material. Besides, the sensitivity of P-HCF pressure sensor can reach 31.92 kPa^−1^, and meanwhile, the linear band of P-HCF pressure sensor can arrive at 24 Pa–720 kPa, which makes it a good choice for sports monitoring. The designed pressure sensor can be used to monitor the foot pressure during running. By analyzing the gait information during running, it can provide data support and strategy improvement for running. This new dual working mode pressure P-HCF sensor will provide a new way for the development of intelligent sports.

**Supplementary Information:**

The online version contains supplementary material available at 10.1186/s11671-023-03788-7.

## Introduction

At present, flexible pressure sensors mainly include piezoresistive sensors and capacitive sensors, which can transform the pressure received into changes in their own resistance and capacitance, thus realizing pressure sensing [1, 2]. Meanwhile, flexible pressure sensing technology is one of the key technologies of intelligent wearable devices, and its development will promote the development of wearable device [3]. The flexible wearable pressure sensing device is mainly used for health detection and motion detection of the human body, and realize the transformation from the human disease treatment mode to the disease prevention mode [4]. It is worth noting that for human motion monitoring, pressure sensors can detect and analyze micro pressure, such as pulse pressure, eye pressure, and muscle movement, especially foot pressure, which is of great significance [5]. Due to the rapid development of functional materials, flexible sensors and integrated power supply systems, people are increasingly interested in wearable electronic devices with human health monitoring functions [6]. According to previous works [7–9], flexible pressure sensors devices have continued to garner interest in the disciplines of prosthetics, health monitoring, and robotics [7–9]. Among these pressure sensors, resistive pressure sensors are preferred due to their simple working mechanism and cost advantages [10]. Resistive pressure sensors can convert the pressure received into the change of its own resistance, thus realizing the pressure sensing. Generally, engraving process is required to prepare materials with micro nano surface structure. Laser engraving uses advanced laser technology for processing, which belongs to non-contact processing, which avoids scratching the workpiece and achieves engraving, cutting and polishing at the same time. However, traditional mechanical engraving must be in contact with the workpiece, and the workpiece needs to be fixed, which will scratch the workpiece and cause waste of materials. Laser engraving has low energy consumption, no noise and no pollution, which is an energy-saving and environmental protection processing method. Thus, traditional engraving methods cause noise pollution and require large energy consumption [11]. Numerous successful ways involving novel materials and better architectures were used to increase pressure sensitivity [12, 13]. Sun et al. have presented a conductive graphite/polydimethylsiloxane (G/PDMS) foam sheet with a stratified surface microstructure that exhibits a 245 kPa^−1^ sensitivity [14]. Numerous materials have been researched for use in pressure sensors, including conducting polymers, graphite, and nanomaterials, such as carbon nanotubes (CNTs), graphite, and carbon nanofibers (CF) [15–21]. Additionally, degradation efficiency may be enhanced by developing microtomes and microcones on the surface of the material and by generating microscopic and nanoscale micropores inside the material [22, 23]. However, when subjected to high voltage, the compressibility of the material decreases, leading to the saturation of the number of conductive paths and contact areas in the withstand voltage and contact type components, resulting in reduced sensitivity and irregular response.

Consequently, flexible sensors have historically struggled to achieve acceptable sensitivity in the high voltage range [24]. The linear sensitivity of a sensor is defined as the relationship between its output signal and the applied load. In practical application, the pressure sensor must work within the linear sensitivity range, which maximizes the cost efficiency and eliminates the need for further signal processing to receive correct information [25–27]. Flexible pressure sensors, on the other hand, can be easily integrated with existing signal conditioning systems on the market, which means they have more market potential in industries like artificial intelligence, healthcare, and more. However, few papers describe sensors with linear sensitivity in the whole spectral resolution range [28]. Researchers are continuing to work on extending the band and responsiveness of linear sensing. Cho et al., for example, developed a surface using reduced graphene oxide (rGO) and an array of PDMS microdome structures [29]. The skin has a sensitivity of 8.5 kPa^−1^. Wong et al. transformed a stiff non-conductive bulk material into a flexible conductive carbon/silicon composite for use as a sensing material, achieving 10.7 kPa^−1^ across a broad pressure range (up to 100 kPa) [30]. Yang et al. recently revealed a pressure sensor with a layered microstructure with an even better sense of 15.4 kPa^−1^ across a broad linear range of over 200 kPa [31]. Although the comparative study has obtained more favorable results, pressure sensors still fail to meet the standards for practical application within the high pressure range. To expand the application range, it is essential to maintain high sensitivity while expanding the linear detection range.

Here, we propose a hybrid conductive elastomeric film oxide-based material with a concave-shape micro-patterned array (P-HCF) on the surface that sustainably shows the necessary sensing qualities with an excellent linear sensitivity of 31.92 kPa^−1^ throughout a wide pressure range of 24 Pa–720 kPa, as well as the ability to sense pressure changes in real time. In this design, PDMS acts as a flexible dielectric in the P-HCF flexible pressure sensor device. When the device is subjected to pressure, the PDMS layer will deform and then cause the pressure device to generate different sensing signal. MXene (MX) can be used as adsorbent due to its unique layered structure, high specific surface area, excellent surface hydrophilicity and rich active sites. The one-dimensional carbon fibers (CF) is introduced into PDMS as a conductive material to enhance its conductivity. As PDMS is hydrophobic, MX needs to be added as adsorbent to fuse CF and PDMS. Compared with the previous sensor materials, we introduced MX into the dielectric elastomer of the sensor, which greatly enhanced the distribution uniformity of conductive materials inside the dielectric elastomer because MX has good adhesion. Unlike conventional flexible detectors, which rely on changes in surface structure to achieve linear sensitivity, this sensing material starts from the surface micro-nano texture characteristics by forming a micro-nano hybrid framework from a multidimensional carbon material in P-HCF by forming a concave-shape micro-pattern that mimics the structure of a human fingerprint. From a macro viewpoint, the array provides multi-step sensing as well. According to the results, the pressure sensor works very well and has the potential for running motion monitoring.

## Materials and methods

### Preparation of P-HCF film

**Fig. 1:**
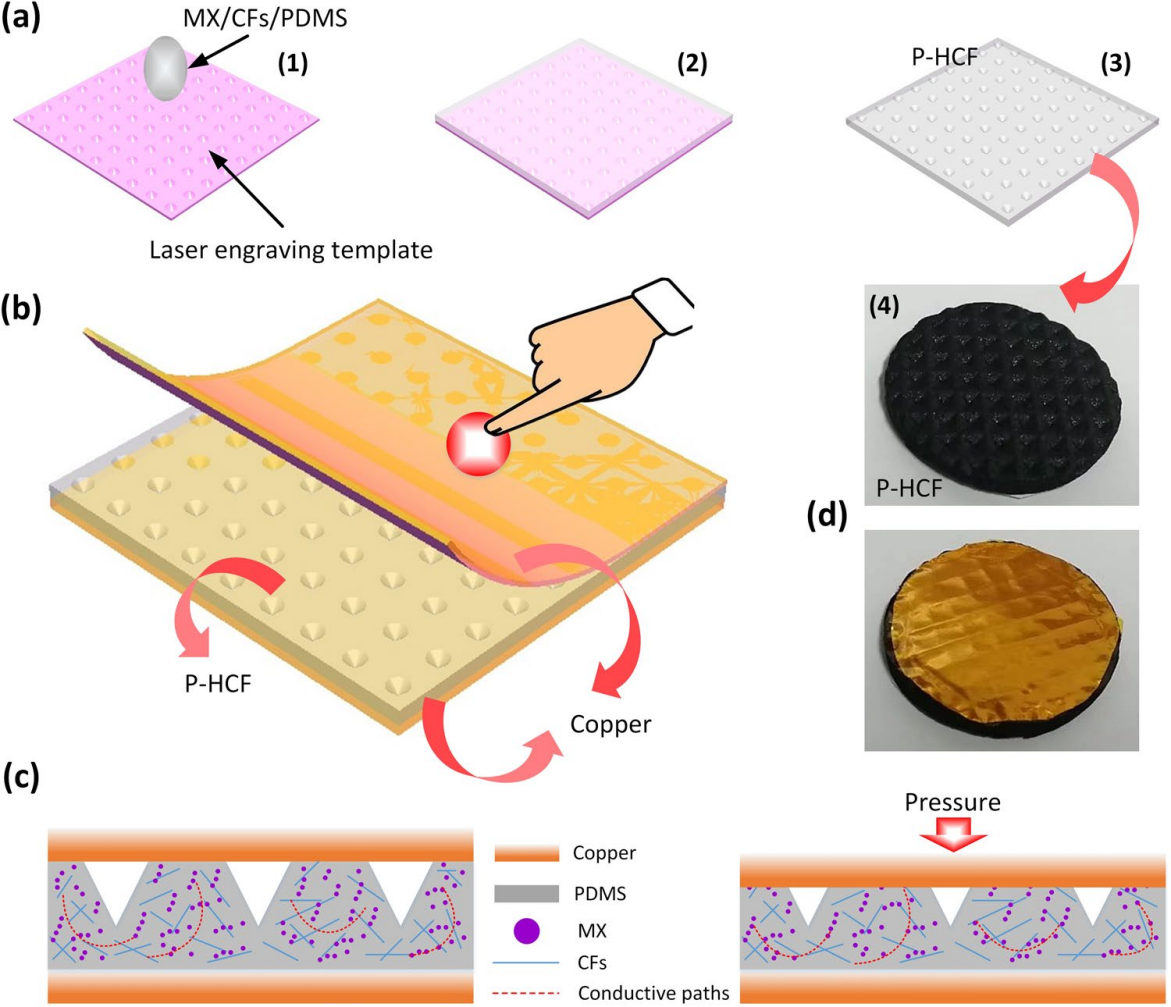
**a1**–**a3** Schematic of the P-HCF preparation process. **a4** The picture of the P-HCF device. **b** Schematic of flexible P-HCF pressure sensor. **c** The basic working principle of the P-HCF pressure sensor. **d** The picture of the P-HCF pressure sensor device

The P-HCF was fabricated by molding the liquid mixture of conductive elastomers on templates. CFs (8 m diameter, 10–100 m length) (400 mesh, Haotian Nano Technology Company) and PDMS (Dow Corning Sylgard 184; the weight ratio of base to curing agent was 10:1) were mixed by a planetary centrifugal mixer. Following that, MX (CCI, Jujo Chemical Co., Ltd. Shenzhen, China) was added to the CFs/PDMS and mixed for 3 h at 400 rpm using an overhead stirrer (IKA, RW20). As shown in Fig. 1a1–a3, the conductive elastomer was bladed onto patterned quartz templates, which were engraved by laser flash (Speedy 360, 60 W) based on programmable patterning with different spacings and depths. The spacings of micro-patterns were 200 μm, 300 μm, and 400 μm; and the depths were 25 μm, 55 μm, 80 μm and 110 μm. The HCF film containing micropatterns were evaporated to dryness in a vacuum oven, cured completely at 100 °C, and then peeled away from the template. Besides, the mass fraction of CF and MX is 2% and 5%, respectively.

### Characterization of P-HCF pressure sensor

The square P-HCF is adhered to the bottom copper laminate electrode using conductive silver glue with the patterned surface facing up. Then, the elastomer top is covered with copper foil to form the pressure sensor. The electrical responses of the sensors were measured by using the mechanical performance testing system (MTS E43.104) and a digital source meter (Keysight B2902A). The applied voltage for measurements of all electrical properties was 0.05 V DC. The curves of pulse monitoring and pressure array were acquired by source meter and electrochemical workstation (Metrohm, M204), respectively.

## Results and discussion

Conductive elastomeric films comprising micro-and nano-hybrid carbon materials with curved concave-shape micro patterned arrays (P-HCFs) can be generated simply and reliably, as seen in Fig. 1a. Figure 1a4 shows the picture of P-HCF device. Besides, Fig. 1b illustrates the layout of the flexible pressure sensing element. P-HCF is sandwiched between two copper electrodes composed of copper clad plates. The pressure sensor's fundamental operation is shown in Fig. 1c. The external pressure changed the contact area between the concave-shape micro-patterned array on the surface and the upper electrode, leading to the decrease in contact resistance. The conductive electrode is copper foil, which is mainly used as two plates of capacitance to conduct the capacitance change of the pressure sensor. Meanwhile, the contact resistance formed between copper electrode and P-HCF film is also an important factor of feedback pressure. Correspondingly, the total current of P-HCF sensor rose with the increase in applied pressure. Moreover, the photograph of the P-HCF pressure sensor is illustrated in Fig. 1d.

**Fig. 2:**
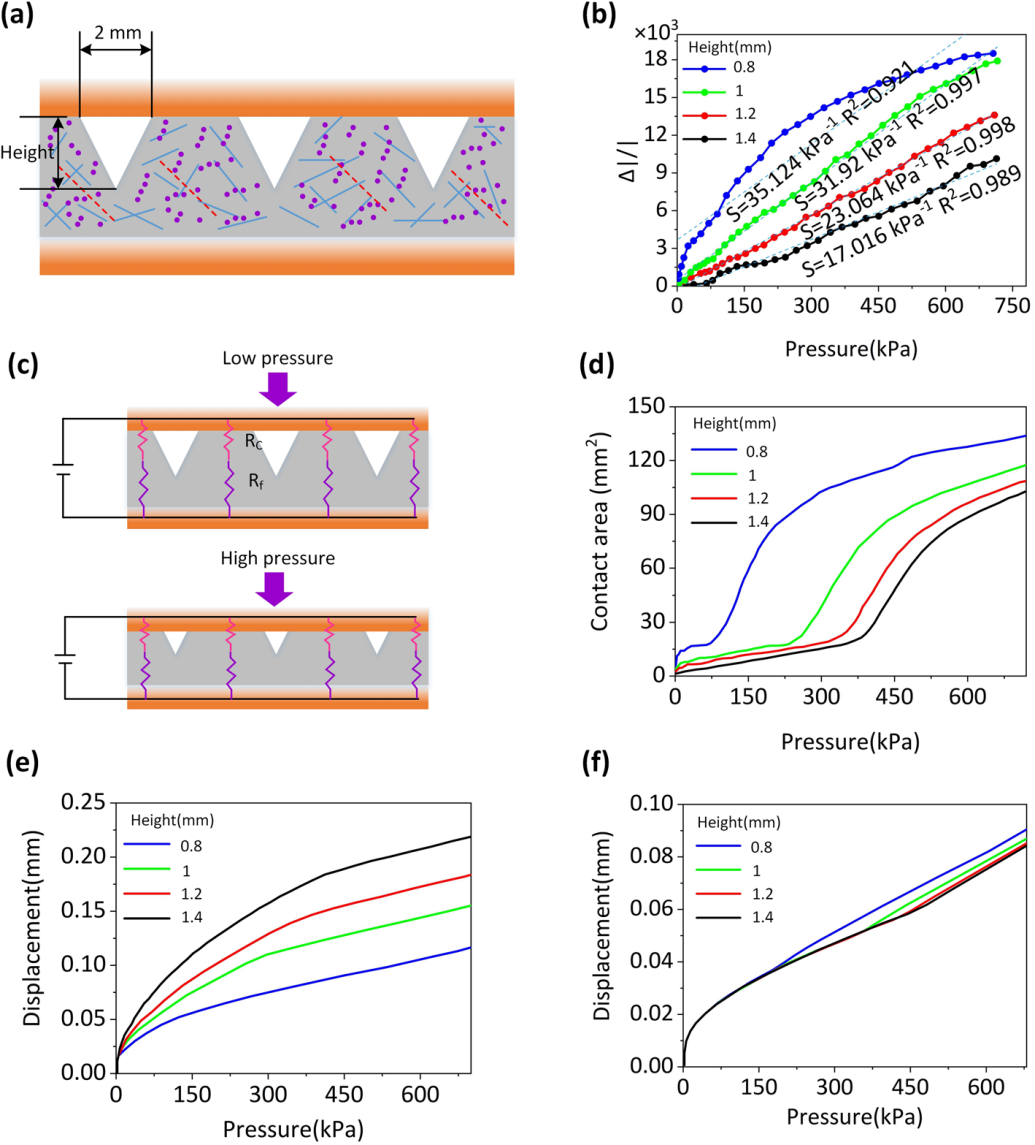
**a** The dimension diagrammatic sketch of P-HCF pressure sensor. **b** 3D surface profiles of HCFs (1: P_0.8_-HCF, 2: P_1_-HCF, 3: P_1.2_-HCF, 4: P_1.4_-HCF). **c** The working mechanism of P-HCF pressure sensor. Change of contact area **d**, total deformation **e**, and deformation of bulk films **f** of P-HCFs in response to different pressure

To show the influence of curved microstructure height on the sensitivity and linear band of flexible pressure sensors, four distinct P-HCFs (P_0.8_-HCF, P_1_-HCF, P_1.2_-HCF, and P_1.4_-HCF) with varying pattern heights were produced while maintaining a pattern spacing and size of 2 mm, as shown in Fig. 2a. When applying pressure to the pliable pressure sensor shown in Fig. 2b, the sensor's performance is demonstrated. The pressure sensor's sensitivity (*S*) is described as *S* = (Δ*I*/*I*)/Δ*P* where Δ*I* and Δ*P* note the measured current and pressure changes, respectively, and *I* denotes the starting current without load. According to the data, as the height of the projectionettes increases, the standardized change in current increases as well (Δ*I*/*I*) eventually approaches a linear relationship with the applied pressure across the whole range of 24 Pa–720 kPa, as shown in Fig. S1 of Supporting Information. Furthermore, the working mechanism of P-HCF pressure sensor is composed of two components: contact resistance (*R*_c_) and film resistance (*R*_f_), as illustrated in Fig. 2c. In detail, different pressures will lead to different degrees of deformation of P-HCF pressure sensor. As shown in Fig. 2d, when the applied pressure grows, the sensor's contact surface will also increase. An electron tunnel junction is formed by sandwiching a thin insulating layer between two metal conductors. It is found that electrons can pass through the tunnel junction, that is, electrons can pass through the insulating layer, which is the tunnel effect. Contact effect is an electrical effect caused by two different materials when they are in contact, which is different from that when they are not in contact and exist independently.

As the pressure load increases, the change in *R*_f_ due to tunneling and contact effects between the internal conducting filler and the body block caused by pattern deformation progressively rises, increasing the sensor's sensitivity in conjunction with *R*_c_. Until the pattern becomes flat, the change in resistance is primarily determined by the *R*_f_ caused by the compressive deformation. As the micropattern's height grows, the process of micropattern to flatten the bulk film takes more time and changes shape more, as shown in Fig. 2f. As a consequence, the P1-HCF sensor's sensitivity diminishes and tends to saturate at high pressure, while sensors with micro-patterns of various heights exhibit essentially linear sensitivity over the entire detecting range, as shown in Fig. 2b. Additionally, the P1-HCF sensor has a high linear test range of 24 Pa–720 kPa. This suggests that with a sufficiently complex micro-pattern design, the conductive material compensates for the contacts' saturation effect as the pressure rises, allowing for a broad linear detection range. The compressibility of the pressure sensor rises with increasing spacing because the efficient Young's modulus of the micropattern decreases at the same pressure [32]. Enhanced micropattern distortion results in a greater contact area and hence extreme susceptibility. However, micropatterns with a relatively large spacing flatten first, and the sensor's sensitivity drops as pressure increases, resulting in a loss of linearity over the whole sensitivity range. On the other hand, it was discovered that sensors with densely separated micropatterns display linear sensitivity across a large sensing area owing to the synergistic impact of contact resistance and film resistance formed during sensing. In the end, the best material to use for a pressure sensor was P1-HCF with a pitch and size of 2 mm [33]. Compared to previous work [15, 34, 35], the pressure sensor we designed can provide larger pressure test range, but the sensing sensitivity is still limited.

**Fig. 3:**
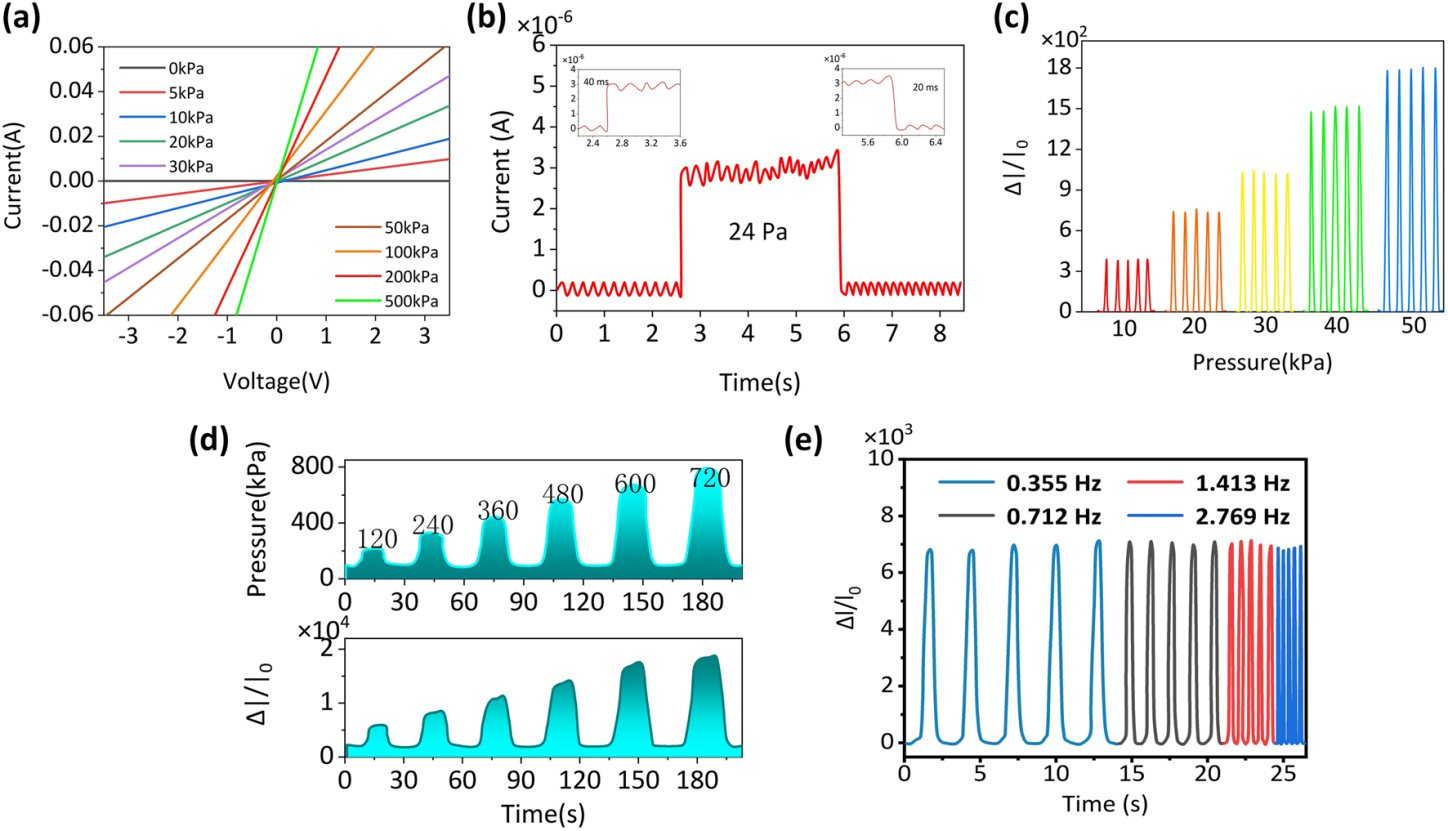
**a** The I–V curves of the P-HCF pressure sensor. **b** Current responses to loading/unloading 24 Pa on the sensor. Inserts: the response time and recovery time. **c** Cyclic current responses of the P-HCF pressure sensor under different pressures. **d** Real-time pressure monitoring of the P1-HCF sensor. **e** Cyclic current responses of the sensor to 240 kPa under different frequencies

The P1-HCF sensor's current–voltage (I–V) curve was tested at various pressure levels ranging from − 3.6 to 3.6 V. We observed a clear linear connection and a consistent response, showing that the ohmic properties are stable (Fig. 3a). The resistance (slope of the curve) reduces proportionately as the applied load rises. To determine the sensor's detection limit (LOD), the present response to stress was established by the following: inserting and lowering a 0.24 g, demonstrating that the sensor is capable of tracking minute pressure fluctuations up to 24 Pa, as seen in Fig. 3b. The inset shows the sensor's immediate sensing reaction time (40 ms) and recovery time (20 ms), indicating that the sensor is fast and equivalent to human skin pressure when exposed to external pressure. As present in Fig. 3c, the sensor is capable of achieving rapid and linear current changes across a modest pressure range. As shown in Fig. 3d, it is worth noticing that the response signal exhibits a strong linear association with the pressure level as the pressure increases in the high-pressure range (120–720 kPa), which is better than previous work [1, 36, 37]. The P1-HCF sensor's response at varied compression frequencies with a constant pressure load of 240 kPa is shown in Fig. 3e. The pressure sensor is shown to react frequently at various compression frequencies. Furthermore, the reliability test and reversibility test of pressure sensor device performance are shown in Figs. S2 and S3 of Supporting Information. From the results, the pressure sensor device has good stability.

**Fig. 4:**
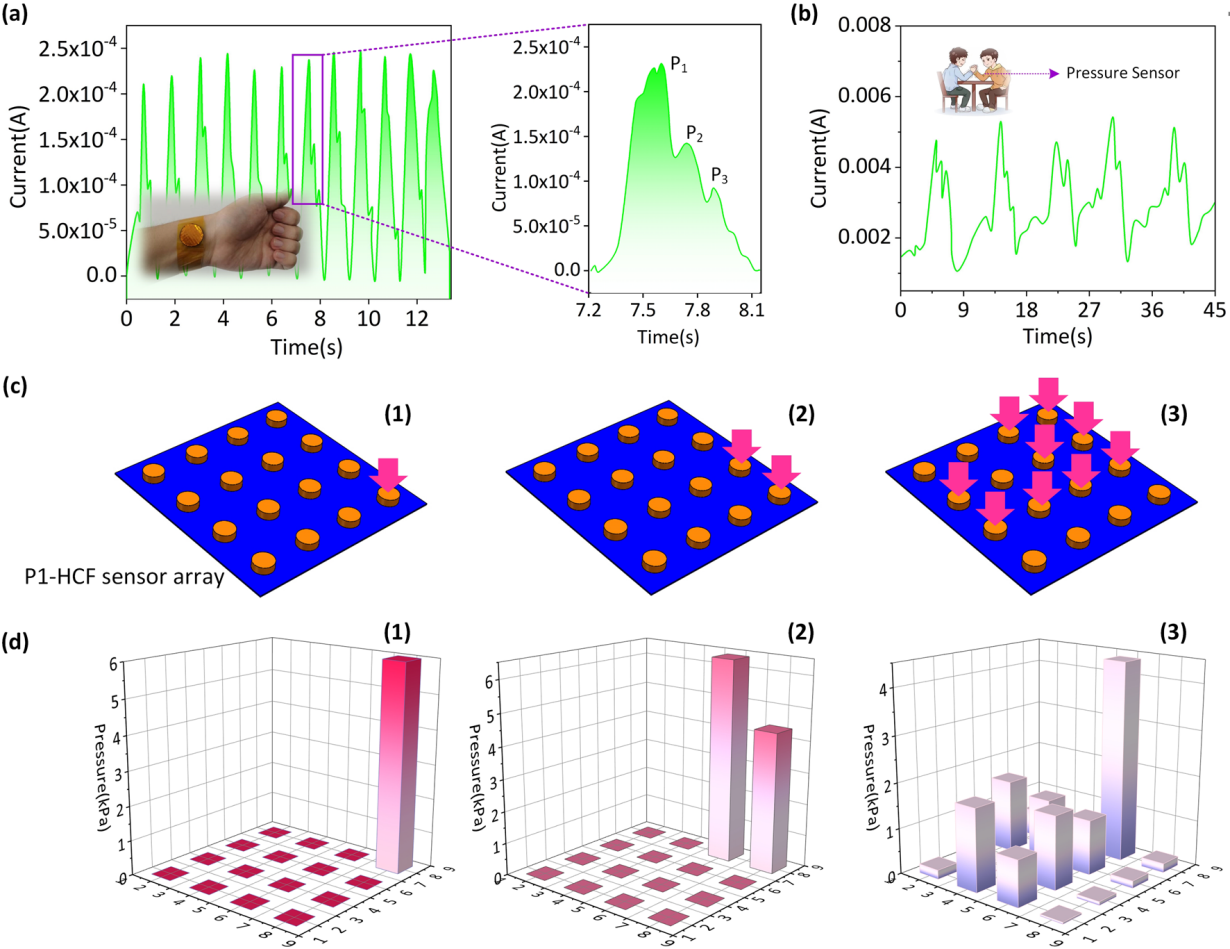
**a** Flexible P_1_-HCF pressure sensor attached on the wrist for pulse signals monitoring. **b** Flexible P1-HCF pressure sensor fixed on the biceps for muscle response monitoring. **c1**–**c3** Schematic of the pressure sensor array assembled from the P_1_-HCFs with 4 × 4 working units. **d1**–**d3** Finger and palm contact schematics with associated pressure maps on the pressure sensor array

To assess the possibility of pressure sensors being used in the diagnosis of cardiovascular illnesses, sensors were worn on the wrist, and the arterial pulse was measured in real-time, as shown in Fig. 4a. A pulse rate of 70 beats per minute was measured here, which is the rate of a healthy adult [38]. The usual arterial waveforms such as shock wave (*P*_1_), tidal wave (*P*_2_), and diastolic wave (*P*_3_) may be recognized without signal amplification or post-processing. Parkinson's disease (PD) is a neurodegenerative illness that is most often diagnosed in middle-aged and older adults, and muscular stiffness is a common symptom of PD patients. Passive motor resistance in flexors and extensors is a common complaint throughout the range of action. Parkinson's patients mainly show static tremor, slowing down and muscle rigidity. Their gait has certain characteristics, mainly showing flustered gait. With the progress of the disease, their gait abnormalities become more obvious. Muscle stiffness is determined in clinical practice by passive limb movement at the joint. Figure 4b depicts a patient's simulated muscular resistance during passive motion with the sensor connected to the biceps muscle, and depicts the accompanying current changes, suggesting that the P_1_-HCF sensor has potential application value in detecting Parkinson's disease. Additionally, the spatial distribution and pressure values may be seen by including a flexible pressure sensor in the sensing array. Figure 4c1–c2 illustrates a 4*4-telescopic pressure sensor array comprised of a P1-HCF sensor and a flexible telescopic circuit that fits inside a hemispherical mold, illustrating that telescopic sensing arrays may be adapted to complicated surfaces in real applications. 3D mapped pictures show pressure values and locations on fingers and palms by height and position, as shown in Fig. 4d1–d3. The sensor can now detect the pressure distribution and the general contour of the object.

**Fig. 5:**
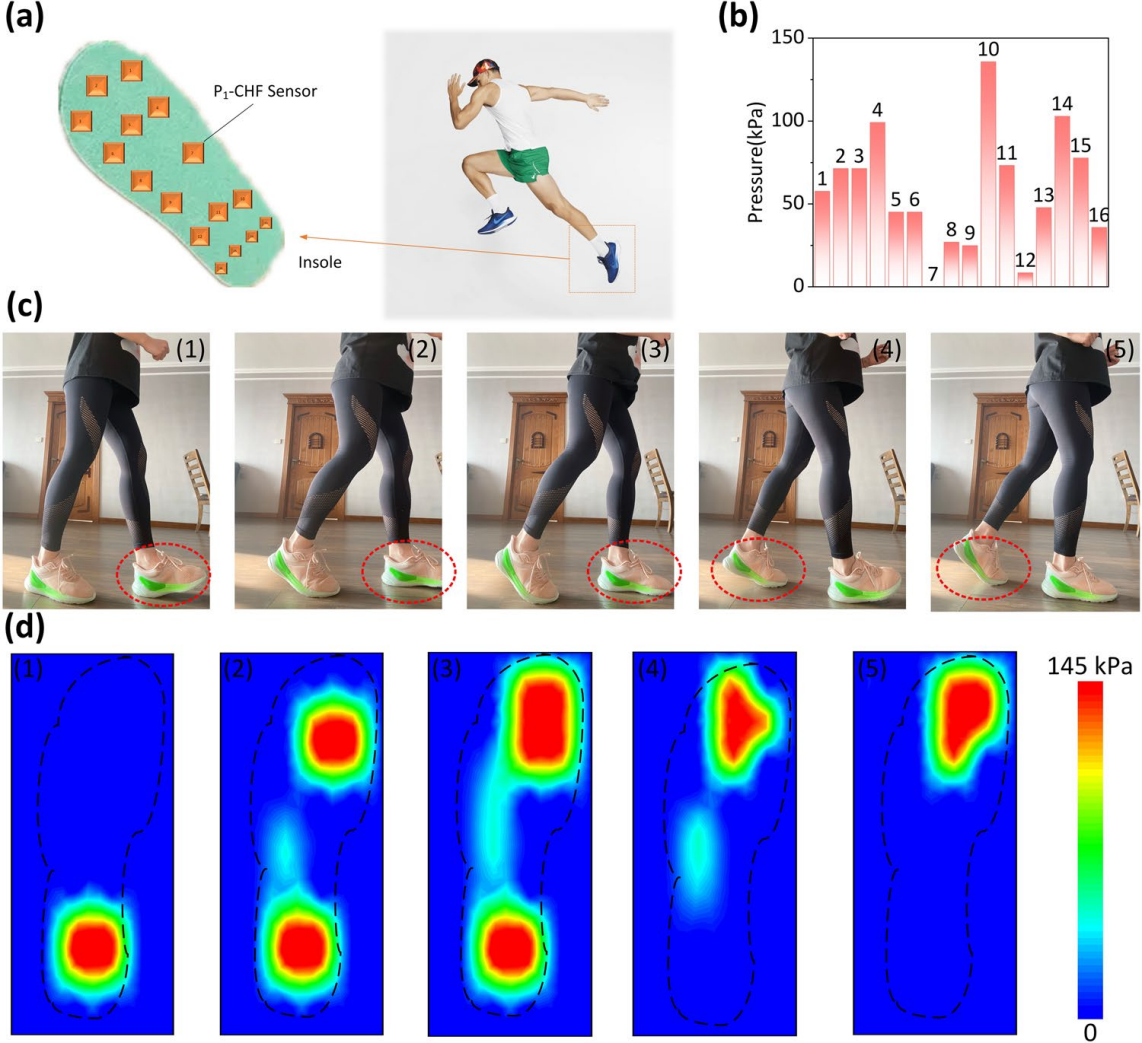
**a** Schematic of the intelligent insole consisting of 16 working sensors. **b** The intelligent insole measures the distribution of plantar pressure throughout the running process. **c1**–**c5** Photographs of the running motion. **d1**–**d5** The change of plantar pressure can be observed during running

To monitor plantar pressure effectively, linear sensing capacity across a wide range of up to several hundred kilopascals is required, as is high sensitivity. As a result, we designed a flexible smart insole with 16 working units located at the toes, forefoot, lateral, and rearfoot, as shown in Fig. 5a. The foot pressure distribution of a person weighing 60 kg in a standing posture is shown in Fig. 5b. The flexible sensors' broad linearity and excellent sensitivity enable them to gather pressure correctly at each spot. Additionally, the pressure distribution changes during running motion were rebuilt using sixteen consecutive sensors, as shown in Fig. 5c1–c5. According to the experimental results shown in Fig. 5d1–d5, the change of plantar pressure can be observed during running. When the human body runs, the forefoot and heel are the main force bearing parts.

## Conclusions

In conclusion, a flexible P-HCF pressure sensor was reported. The multi-step sensing technique enables an extensive dynamic range and great linear sensitivity. The sensing material P-HCF enables the fabrication of the hybrid structured conductive films with concave-shape micro-patterned arrays. 1D CF and 2D MX are used as conductive fillers in PDMS, resulting in a multi-step sensing structure at the microscale network. Filler substrate elastomeric materials have a greatly improved detection range and responsiveness as a result of this improvement. Besides, the sensitivity of P-HCF pressure sensor can reach 31.92 kPa^−1^, and meanwhile, the linear band of P-HCF pressure sensor can arrive at 24 Pa–720 kPa, which makes it a good choice for pressure testing applications. This sensor may be used to detect human pulses and aid in the diagnosis of Parkinson's disease. Simultaneously, it may be useful for monitoring human gait and determining the distribution of foot pressure. This research combines multifunctional material technology with flexible sensing technology, which provides a new idea for the running motion monitoring.

## Supplementary Information

**Additional file 1.**

## Data Availability

All data and materials are available without restrictions.
