# Supplementary material for: A wearable electronic based on flexible pressure sensor for running motion monitoring
Source: Discov Nano. 2023 Mar 1;18(1):28. doi: 10.1186/s11671-023-03788-7 (PMC9978046; doi:10.1186/s11671-023-03788-7)
Supplement: Supplementary file 1 — Additional file 1. [file 11671_2023_3788_MOESM1_ESM.doc]

**A Wearable Electronic Based on Capacitive Flexible Pressure Sensor for Running Motion Monitoring**

Xiaoming Chang^1*^

^1^Physical Education College of Harbin Normal University, Harbin, Heilongjiang Province, 150001, China.

^*^Correspondence: xiaoyang@hsdtky.ntesmail.com


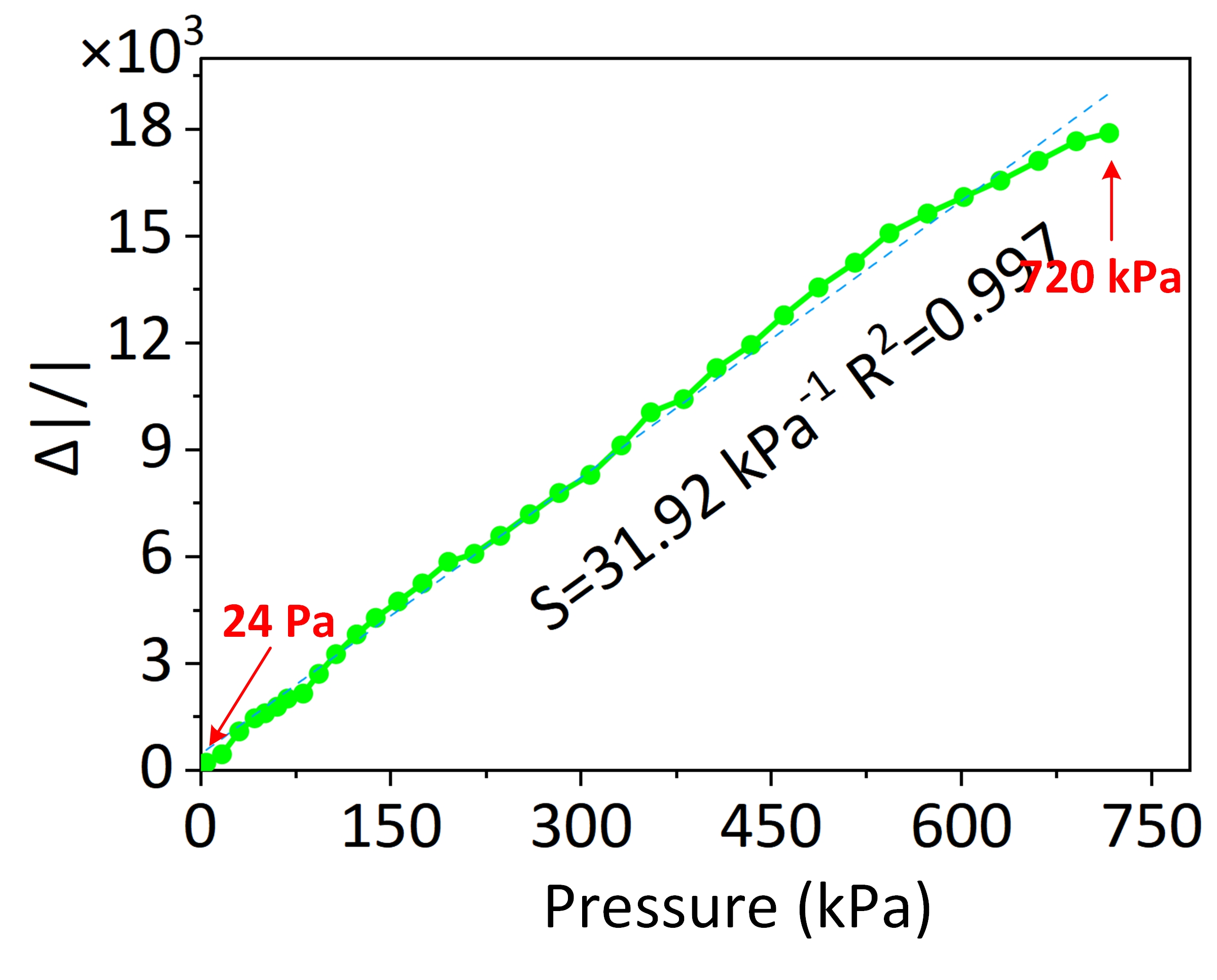


Fig. S1. 3D surface profiles of HCFs (P_1_-HCF).


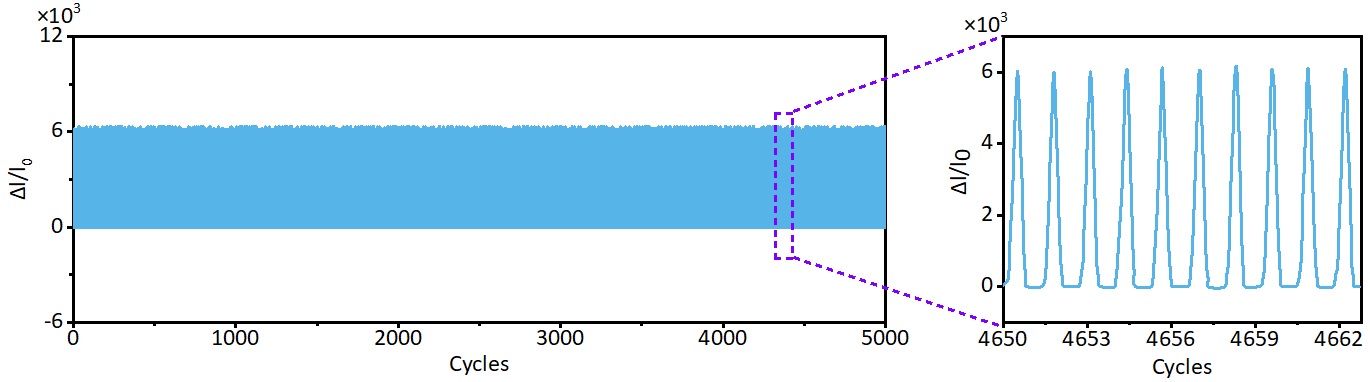


Fig. S2. Reliability test of the pressure sensor device.


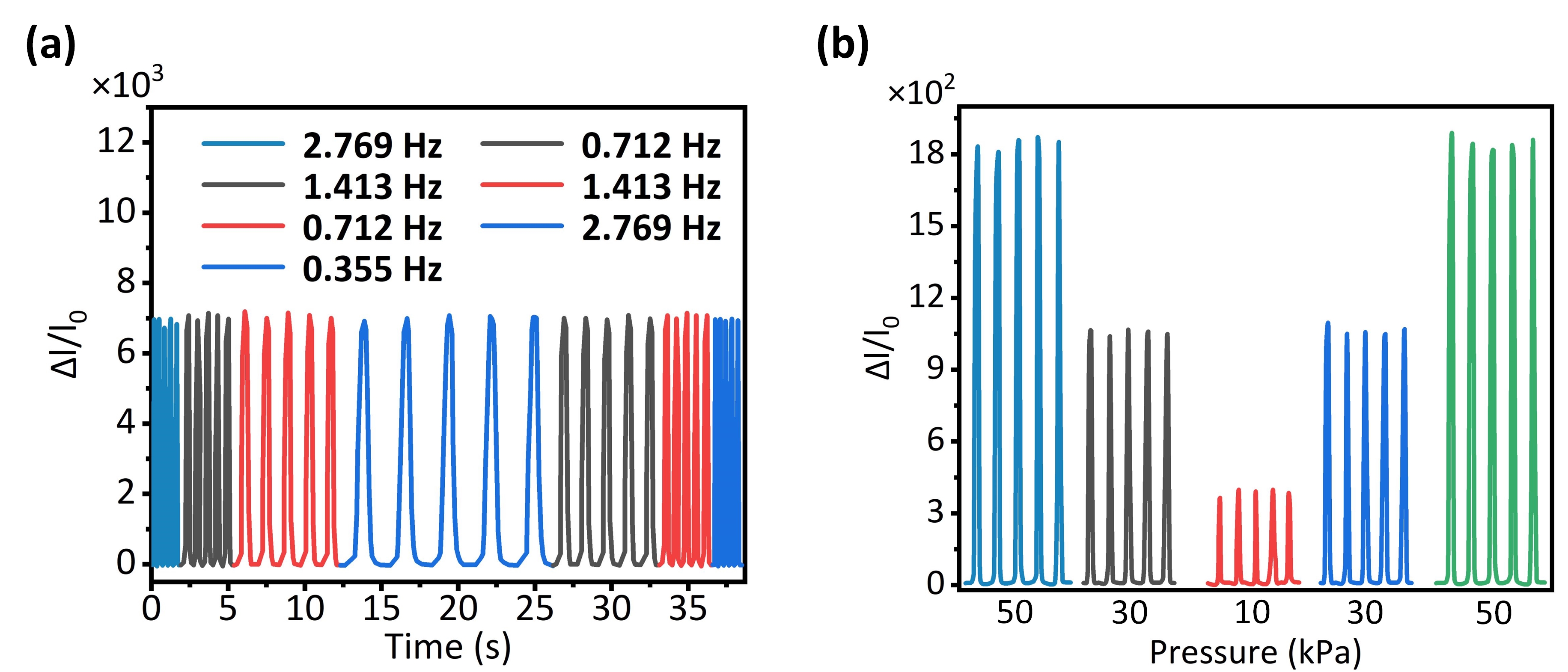


Fig. S3 (a, b). Reversibility test experiment of the pressure sensor device.
